# Association of the number of teeth and self-rated mastication with self-rated health in community-dwelling Japanese aged 40 years and older: the Yamagata cohort study

**DOI:** 10.1038/s41598-022-25690-5

**Published:** 2022-12-05

**Authors:** Shigeo Ishikawa, Tsuneo Konta, Shinji Susa, Kenichi Ishizawa, Naohiko Makino, Yoshiyuki Ueno, Naoki Okuyama, Mitsuyoshi Iino

**Affiliations:** 1 Department of Dentistry, Oral and Maxillofacial Plastic and Reconstructive Surgery, Faculty of Medicine, Yamagata University, 2-2-2 Iida-Nishi, Yamagata, 990-9585 Japan; 2 Department of Public Health and Hygiene, Yamagata University Graduate School of Medicine, 2-2-2 Iida-Nishi, Yamagata, 990-9585 Japan; 3 Department of Neurology, Hematology, Metabolism, Endocrinology and Diabetology, Faculty of Medicine, Yamagata University, 2-2-2 Iida-Nishi, Yamagata, 990-9585 Japan; 4 Yamagata University Health Administration Center, Yamagata, Japan; 5 Global Center of Excellence, Yamagata University School of Medicine, 2-2-2 Iida-Nishi, Yamagata, 990-9585 Japan

**Keywords:** Health care, Dentistry

## Abstract

Self-rated health (SRH) is a predictive factor for health-related prognoses such as mortality. This study aimed to comprehensively investigate the risk factors for poor SRH in the general population of Japan, focusing on the combination of the number of teeth and self-rated mastication. Individuals aged at least 40 years in Yamagata Prefecture, Japan, were surveyed from 2017 to 2021. The participants answered a self-administered postal survey on lifestyle factors, medical history, physical and mental conditions, oral health, and dietary intake, and 6739 participants were included. Multivariate logistic regression analysis showed that individuals with less than 20 teeth and who bite tightly on one side or neither side were at a 1.422- and 1.952-fold significantly higher risk, respectively, of poor SRH than individuals with at least 20 teeth and who bite tightly on both sides. Moreover, individuals who had less than 20 teeth but could bite tightly on both sides did not have a significant risk compared to those who had at least 20 teeth and could bite tightly on both sides. Regarding individuals with more than 20 teeth, there was no difference between those who could and could not bite tightly on both sides, although the odds ratios for poor SRH tended to increase for those who could bite on one side or neither side. Our results emphasize the importance of having at least 20 teeth without periodontal disease and oral rehabilitation using a type of prosthesis for SRH, even with less than 20 teeth.

## Introduction

Self-rated health (SRH) refers to self-awareness of one’s general health and is easily assessed with the simple question, “How would you rate your general health?”^1^. Although objective data such as blood pressure, body mass index (BMI), and blood chemical analysis are valuable for predicting health prognosis, subjective data such as SRH are well known to predict health prognosis^1–4^. Furthermore, recent prospective cohort studies have accumulated high-quality evidence that SRH is one of the predictive factors for health-related prognoses such as mortality^2,5–7^. Therefore, SRH is one of the recommendations by the World Health Organization for health monitoring^2^. In Japan, the SRH index has been valued and included as an important question in the comprehensive survey of living conditions, which is conducted routinely by the Ministry of Health, Labour, and Welfare for all households and their members within a stratified random sampling area from the census zone^8^.

Risk factors for poor SRH have been assessed in previous studies. Physical or mental conditions, psychosocial factors, and socioeconomic status, including educational status, physical activity, smoking, drinking habits, BMI, and quality of sleep, have been discussed extensively in relation to SRH^9–19^. Furthermore, the associations between SRH and oral health status, such as the number of teeth, have been investigated^8,20–24^. The lower the number of teeth, the poorer the SRH^8,20–22^, and the higher the self-rated mastication, the higher the SRH satisfaction^8,23,24^. However, to the best of our knowledge, factors such as number of teeth and self-rated mastication have been investigated separately and independently^8,20–24^, and there have been no reports on the association of the number of teeth and self-rated mastication with SRH in Japan. In the case of tooth loss, which leads to mastication problems, mastication restoration is possible through appropriate prosthesis repair^25,26^, which should contribute to a higher SRH. To survey the risk factors for SRH regarding oral health, details of the combination of the number of teeth and self-rated mastication status should be investigated. It is not sufficient to assess the number of teeth alone as a risk factor for SRH. Furthermore, to the best of our knowledge, almost all studies on the association between SRH and oral health have been performed in individuals older than 60 years^20,21,23,24^. Very few studies on a wider age range were found^8^, and the authors did not sufficiently consider well-known risk factors for poor SRH, such as mental status, exercise, and local community participation^8^. To accurately survey the risk factors for poor SRH regarding oral health, well-reported risk factors and a wide age range of study participants should be considered.

Therefore, we conducted a cross-sectional study using data from a community-based cohort study, the Yamagata Study, to survey the risk factors for poor SRH. We hypothesized that there is an association of poor SRH with having fewer remaining teeth and poor self-rated mastication and of good SRH with having fewer remaining teeth and good self-rated mastication, which indicates that mastication is restored by the use of a prosthesis.

## Materials and methods

### Study design and participants

This study was performed as part of an ongoing molecular epidemiological study utilizing the regional characteristics of the 21st Century Centers of Excellence Program in Japan. This study was a community-based and design-incorporated baseline survey conducted using a self-administered questionnaire.

The survey population in this study was the general population aged at least 40 years in Yamagata Prefecture, Japan. The 8783 participants of Yamagata Prefecture received a postal survey, which was a self-administered questionnaire from 2017 to 2021. The questionnaires were completed by 7447 individuals (2558 men and 4889 women), 708 of whom were excluded and 6739 were included in the final statistical analysis. Data of 708 participants were excluded because of incomplete answers on SRH, number of teeth, and self-rated mastication.

### Measurements

The questionnaire included questions pertaining to basic characteristics such as sex, age, BMI, and marital and educational status, lifestyle, physical and mental conditions, medical history, and oral health. The details have been described below.

#### Basic characteristics

We classified the basic characteristics of participants as follows:

- Age: 40–49, 50–59, 60–69, and 70–79 years.
- BMI: < 18.5, 18.5–24.9, 25–30, and > 30 kg/m^2^.
- Marital status: never married, married, divorced, separated, bereaved, and other.
- Educational status: university graduate or above, high school/junior college/professional training college/university drop-out, and junior high school graduate.

#### Lifestyle

Factors affecting lifestyle were categorized for appropriate analysis as follows:

Frequency of drinking habit: never (including those who had quit drinking), rarely, one, two, or three times a month, one or two times a week, three or four times a week, five or six times a week, and every day.

- Smoking habits: current smokers and never smokers.
- Exercise routine: every day, three or four times a week, one or two times a week, one, two, or three times a month, and less than once a month.
- Duration of sleep: five, six, seven, eight, nine, and ˃ 10 h.
- Frequency of local community participation: more than once a week, less than once a week, sometimes, and never or rarely.

#### Physical and mental conditions

The physical condition was evaluated in the form of SRH, which was assessed using a single question, “On the whole, how would you rate your general health in the past month?” The five possible answers were excellent, very good, good, fair, and poor, which were further categorized into two groups: excellent/very good/good and fair/poor. The mental condition was evaluated in the form of frequency of feeling depressed in the last week, which was classified into four categories: never, occasionally, often, and always.

#### Medical history

The medical history of the patients, especially pertaining to cancer, cardiovascular disease, and other diseases, was assessed and classified as follows:

Cardiovascular disease: myocardial infarction, cardiac angina, cerebral stroke including brain hemorrhage, brain infarction, subarachnoid hemorrhage, cardiac failure, atrial fibrillation, hypertension, or other cardiovascular diseases (free description).

Other diseases: diabetes mellitus, hyperlipidemia, gout, asthma, chronic obstructive pulmonary disease, chronic bronchitis, chronic renal failure including undergoing renal dialysis, cataract, glaucoma, gastric polyp, colonic polyp, gastric ulcer, duodenal ulcer, chronic hepatitis, hepatic cirrhosis, gallstones, ureteral stones, kidney stones, sleep apnea, depressive illness, and fracture (waist, arm or wrist, and femoral), excluding fractures caused by traffic, fall, or work-related accidents.

#### Oral health

Oral health was assessed in the form of number of teeth and self-rated mastication using two questions, “How many teeth do you have? (Restored teeth and teeth with posts and crowns were counted, but dental implants were not)” and “Can you bite tightly on both sides with your own teeth or prostheses?” The three possible answers for self-rated mastication were able to bite tightly on both sides, one side, and neither side.

### Statistical analyses

The distribution of characteristics was analyzed using the chi-square test for categorical variables. Crude odds ratio (OR) for the risk of poor SRH (categorized as fair/poor vs. excellent/very good/good) was calculated using univariate logistic regression analysis for each variable. To examine the independent association between SRH and analyzed categorical parameters, we performed multivariate logistic regression analysis using the backward stepwise method to estimate the adjusted OR and 95% confidence interval (95% CI). We selected representative variables that were significant in the univariate analysis (p < 0.05) to be included in the multivariate regression analysis. Statistical significance was set at a p-value of < 0.05. All statistical analyses were performed using SPSS version 25.0 (IBM Corp., Armonk, NY, USA).

### Ethical approval

All procedures performed in this study involving human participants were in accordance with the ethical standards of the institutional and/or national research committee and with the 1964 Helsinki declaration and its later amendments or comparable ethical standards. The ethics committee of Yamagata University approved this study protocol (2022-106).

### Informed consent

Informed consent was obtained from all participants in the original cohort study. In this study, the participants were given an option to opt out online. None of the patients declined to participate.

## Results

The distributions of the parameters of participants in each group for SRH (excellent/very good/good or fair/poor) are shown in Table 1. The significantly different parameters were age, BMI, the combination of number of teeth and self-rated mastication, marital status, frequency of drinking habit, exercise routine, frequency of feeling depressed, sleeping hours, frequency of local community participation, and history of cancer, cardiovascular disease, or other diseases.

**Table 1:** Characteristics of subjects.

Variable	Self-rated health				P-value^‡^
	Excellent/Very good/Good		Fair/Poor		
	n	%	n	%	
**Sex**					
Male	1938	34.3	344	31.6	0.093
Female	3714	65.7	743	68.4	
**Age**					
40–49	173	3.1	52	4.8	0.001*
50–59	680	12.1	160	14.8	
60–69	2405	42.9	421	39.0	
70–79	2348	41.9	446	41.3	
**BMI**					
18.5–25	3957	70.9	705	65.5	< 0.001*
< 18.5	323	5.8	115	10.7	
25–30	1161	20.8	219	20.4	
> 30	142	2.5	37	3.4	
**Tooth number and self-rated mastication**					
≥ 20 and bite tightly on both side	3857	68.2	670	61.6	< 0.001*
≥ 20 and bite tightly on one side	450	8.0	114	10.5	
≥ 20 and bite tightly on neither side	89	1.6	29	2.7	
< 20 and bite tightly on both side	900	15.9	164	15.1	
< 20 and bite tightly on one side	227	4.0	64	5.9	
< 20 and bite tightly on neither side	129	2.3	46	4.2	
**Marital status**					
Never married	223	4.0	61	5.7	0.002*
Married	4666	83.3	848	78.9	
Divorced	187	3.3	48	4.5	
Separation	24	0.4	11	1.0	
Bereavement	485	8.7	102	9.5	
Other	18	0.3	5	0.5	
**Educational status**					
University graduate or above	584	10.4	102	9.6	0.439
High school/Junior College/Professional training college/Drop out of University	4479	80.1	853	79.9	
Junior high school graduate	530	9.5	112	10.5	
**Frequency of drinking habit**					
Never (including quit drinking)	2497	44.2	549	50.5	0.002*
Rarely	285	5.0	56	5.2	
1 or 2 or 3 times a month	504	8.9	102	9.4	
1 or 2 times a week	504	8.9	80	7.4	
3 or 4 times a week	432	7.6	71	6.5	
5 or 6 times a week	415	7.3	57	5.2	
Every day	1015	18.0	172	15.8	
**Current smoker**					
No	5083	93.0	987	93.4	0.741
Yes	382	7.0	70	6.6	
**Exercise routine**					
Every day	1408	26.3	206	20.2	< 0.001*
3 or 4 times a week	1263	23.6	217	21.3	
1 or 2 times a week	985	18.4	204	20.0	
1 or 2 or 3 times a month	696	13.0	152	14.9	
Less than 1 time a month	999	18.7	239	23.5	
**Feeling of depression**					
Never	3710	67.9	372	35.5	< 0.001*
Occasionally	1556	28.5	482	45.9	
Often	156	2.9	137	13.1	
Always	45	0.8	58	2.2	
**Sleeping hours**					
7	2184	38.8	342	31.5	< 0.001*
5	316	5.6	128	11.8	
6	2135	37.9	446	41.1	
8	857	15.2	131	12.1	
9	131	2.3	32	3.0	
≥ 10	10	0.2	5	0.5	
**Local community participation**					
More than one time a week	559	10.1	85	8.0	< 0.001*
Less than one time a week	360	6.5	65	6.1	
Sometimes	2407	43.3	368	34.6	
Never or hardly ever	2233	40.2	546	51.3	
**History of cancer**					
No	5048	89.3	917	84.4	< 0.001*
Yes	604	10.7	170	15.6	
**History of cardiovascular disease**					
No	3751	66.4	687	63.2	0.047*
Yes	1901	33.6	400	36.8	
**History of other disease**					
No	2243	39.7	354	32.6	< 0.001*
Yes	3409	60.3	733	67.4	

*BMI* body mass index.

^‡^p-value based on the chi-squared test.

*Statistically significant (p < 0.05).

Significant variables in the univariate logistic regression analysis for the risk of poor SRH are shown in Table 2. The significant parameters were age, BMI, the combination of number of teeth and self-rated mastication, marital status, frequency of drinking habit, exercise routine, frequency of feeling depressed, sleeping hours, local community participation, and medical history of cancer, cardiovascular disease, or other diseases.

**Table 2:** Crude and adjusted odds ratios and 95% confidence intervals of the variables associated with self-rated health.

	Self-rated health					
	Crude OR†	95% CI	p value	Adjusted OR‡	95% CI	p value
**Tooth number and self-rated mastication**						
≥ 20 and bite tightly on both side	1			1		
≥ 20 and bite tightly on one side	1.458	1.169–1.820	0.001*	1.239	0.965–1.590	0.093
≥ 20 and bite tightly on neither side	1.876	1.224–2.875	0.004*	1.429	0.866–2.357	0.163
< 20 and bite tightly on both side	1.049	0.871–1.263	0.613	1.099	0.884–1.365	0.395
< 20 and bite tightly on one side	1.623	1.215–2.167	0.001*	1.422	1.015–1.992	0.041*
< 20 and bite tightly on neither side	2.053	1.452–2.903	0.000*	1.952	1.265–3.014	0.003*
**Sex**						
Male	1					
Female	1.127	0.981–1.295	0.092			
**Age**						
40–49	1					
50–59	0.783	0.549–1.116	0.176			
60–69	0.582	0.420–0.807	0.001*			
70–79	0.632	0.456–0.876	0.006*			
**BMI**						
18.5–25	1.000			1		
< 18.5	1.998	1.592–2.509	< 0.001*	1.749	1.349–2.268	< 0.001*
25–30	1.059	0.898–1.249	0.498	0.996	0.825–1.204	0.970
> 30	1.462	1.010–2.118	0.044*	1.239	0.805–1.907	0.330
**Marital status**						
Never married	1					
Married	0.664	0.496–0.890	0.006*			
Divorced	0.938	0.613–1.436	0.769			
Separation	1.676	0.778–3.611	0.188			
Bereavement	0.769	0.539–1.096	0.146			
Other	1.015	0.362–2.846	0.977			
**Educational status**						
University graduate or above	1					
High school/Junior College/professional training college/Drop out of Unversity	1.090	0.873–1.362	0.446			
Junior high school graduate	1.210	0.903–1.622	0.202			
**Frequency of drinking habit**						
Never (including quit drinking)	1					
Rarely	0.894	0.661–1.208	0.464			
1 or 2 or 3 times a month	0.920	0.730–1.161	0.484			
1 or 2 times a week	0.722	0.560–0.930	0.012*			
3 or 4 times a week	0.748	0.572–0.977	0.033*			
5 or 6 times a week	0.625	0.467–0.836	0.002*			
Every day	0.771	0.640–0.928	0.006*			
**Current smoker**						
No	1					
Yes	0.944	0.725–1.229	0.667			
**Exercise routine**						
Every day	1			1		
3 or 4 times a week	1.174	0.956–1.442	0.125	1.178	0.941–1.475	0.152
1 or 2 times a week	1.416	1.147–1.746	0.001*	1.436	1.141–1.808	0.002*
1 or 2 or 3 times a month	1.493	1.188–1.876	0.001*	1.459	1.137–1.872	0.003*
Less than 1 time a month	1.635	1.334–2.004	0.000*	1.422	1.132–1.785	0.002*
**Feeling of depression**						
Never	1			1		
Occasionally	3.089	2.665–3.581	< 0.001*	2.949	2.517–3.455	< 0.001*
Often	8.758	6.800–11.280	< 0.001*	7.362	5.588–9.699	< 0.001*
Always	12.854	8.585–19.247	< 0.001*	13.238	8.530–20.544	< 0.001*
**Sleeping hours**						
7	1			1		
5	2.587	2.045–3.272	< 0.001*	1.796	1.367–2.360	< 0.001*
6	1.334	1.145–1.555	< 0.001*	1.128	0.949–1.341	0.172
8	0.976	0.786–1.212	0.827	1.006	0.789–1.282	0.961
9	1.560	1.560–1.043	0.031*	1.739	1.115–2.710	0.015*
≥ 10	3.193	1.085–9.398	0.035*	2.612	0.703–9.702	0.152
**Local community participation**						
More than one time a week	1			1		
Less than one time a week	1.187	0.838–1.683	0.335	1.160	0.796–1.691	0.439
Sometimes	1.005	0.781–1.295	0.966	0.915	0.693–1.209	0.533
Never or hardly ever	1.608	1.257–2.058	< 0.001*	1.242	0.945–1.632	0.121
**History of cancer**						
No	1			1		
Yes	1.549	1.289–1.863	< 0.001*	1.644	1.332–2.030	< 0.001*
**History of cardiovascular disease**						
No	1					
Yes	1.149	1.004–1.315	0.044*	1.161	0.992–1.359	0.063
**History of other disease**						
No	1			1		
Yes	1.362	1.187–1.563	< 0.001*	1.433	1.223–1.681	< 0.001*

*BMI* body mass index, *OR* odds ratio, *CI* confidence interval.

^†^Crude OR for “poor self-rated health (vs. “Excellent”/“Very good”/“Good”).

^‡^Adjusted OR for variables with p < 0.05 in the univariate analysis using backward stepwise method.

*Statistically significant (p < 0.05).

The significant variables in the multivariate logistic regression analysis obtained from the univariate analysis are also shown in Table 2. The independent risk factors for the risk of poor SRH were BMI, the combination of number of teeth and self-rated mastication, exercise routine, feeling depressed, sleeping hours, and history of cancer and other diseases. A BMI of < 18.5 compared to the desirable BMI range was a risk factor for poor SRH (OR = 1.749, 95% CI 1.349–2.268, p < 0.001). Regarding exercise routine, one or two times a week, one, two, or three times a month, and less than once a month compared to every day were risk factors for poor SRH (OR = 1.436, 95% CI 1.141–1.808, p = 0.002; OR = 1.459, 95% CI 1.137–1.872, p = 0.003; and OR = 1.422, 95% CI 1.132–1.785, p = 0.002, respectively). Regarding the frequency of feeling depressed in the past week, occasionally, often, and always compared to never were risk factors for poor SRH (OR = 2.949, 95% CI 2.517–3.455, p < 0.001; OR = 7.362, 95% CI 5.588–9.699, p < 0.001; and OR = 13.238, 95% CI 8.530–20.544, p < 0.001, respectively). Compared to sleeping for 7 h, sleeping for 5 and 9 h were risk factors for poor SRH (OR = 1.796, 95% CI 1.367–2.360, p < 0.001 and OR = 1.739, 95% CI 1.115–2.710, p = 0.015, respectively). Histories of cancer and other diseases were also risk factors (OR = 1.644, 95% CI 1.332–2.030, p < 0.001 and OR = 1.433, 95% CI 1.223–1.681, p < 0.001, respectively). Compared to individuals with at least 20 teeth and who bite tightly on both sides, individuals with less than 20 teeth and who bite tightly on one side or neither side had a 1.422- or 1.952-fold significant higher risk of poor SRH (OR = 1.422, 95% CI 1.015–1.992, p = 0.041 and OR = 1.952, 95% CI 1.265–3.014, p = 0.003, respectively). However, individuals who had less than 20 teeth but could bite tightly on both sides did not have a significant risk compared to individuals who had at least 20 teeth and could bite tightly on both sides. There were also no differences between individuals with at least 20 teeth who could and could not bite tightly on both sides.

## Discussion

In this study, we comprehensively surveyed the risk factors for poor SRH in the general Japanese population and found that compared to individuals with at least 20 teeth and who bite tightly on both sides, individuals who have less than 20 teeth and cannot bite tightly on both sides are at risk of poor SRH. Ideal occlusal rehabilitation with prosthesis might not have been appropriate for the participants with fewer than 20 teeth and who cannot bite on both sides, and the participants might have used incompatible prosthesis. Alternatively, prosthesis rehabilitation might not have been performed for these patients, even if the number of remaining teeth is less. Insufficient occlusal rehabilitation leads to low masticatory ability^27^. Low masticatory ability meant that the food form or type of food consumed by the participants is limited. Food content is an established risk factor for poor SRH^28,29^. Considering the above, our present results must be reasonable.

Furthermore, we found that compared to individuals with at least 20 teeth and who could bite tightly on both sides, individuals with less than 20 teeth and who could bite tightly on both sides were not at risk of poor SRH. Appropriate oral rehabilitation using suitable prostheses may contribute to better SRH, even if the number of remaining teeth is less. Therefore, for better SRH, oral rehabilitation using a suitable prosthesis is important if people lose some teeth, which leads to masticatory dysfunction.

In the present study, individuals with at least 20 teeth but bite tightly on only one side or neither side were not at risk of poor SRH compared to individuals with at least 20 teeth and who could bite tightly on both sides. However, the OR for poor SRH tended to increase for individuals with at least 20 teeth but who could bite tightly on one side or neither side. In this study, an oral examination was not performed, and only a questionnaire on oral health status was administered. Therefore, the actual oral condition was unclear; however, a participant with at least 20 teeth who could not bite on both sides indicated that the participant had periodontal disease. One of the most distinctive symptoms of periodontal disease is tooth mobility. The participants with mobile teeth, associated with periodontal disease, might have answered that they could not bite on both sides even if they had at least 20 teeth. An association between masticatory ability and SRH has already been reported^23,24^. Therefore, the present results are consistent with previous results, although this was only an observed trend that was not statistically significant. Recently, associations between laboratory values and SRH have been investigated^7,30–33^. In particular, attention has been paid to the associations between inflammatory indicators, such as C-reactive protein (CRP) levels, and SRH^7,32,33^. Park et al. reported that poor SRH correlated with low-grade inflammation (high-sensitivity CRP levels) in Korean male adults^33^. Furthermore, Gupita et al. reported that individuals with relatively poor SRH might be aware of underlying inflammation^7^. Periodontal disease is associated with many inflammation-derived subjective symptoms such as oral malodor, non-physiological tooth mobility, gingival bleeding, gingival swelling, and gingival redness. The participant may be aware of an underlying inflammation of periodontal tissue, leading to poor SRH. Furthermore, the association between periodontal disease and CRP levels has already been established^13,34,35^. Considering these results and backgrounds, our present results are understandable.

The strength of the present study is that it is the first study to confirm the association between SRH and the combination of number of teeth and self-rated mastication in the general Japanese population with a wide age range. More teeth or higher self-rated mastication are likely to contribute positively to SRH^8,20–24^. Notably, fewer teeth with a type of inserted prosthesis and higher self-rated mastication contributed to good SRH, which is similar to the contribution of more teeth and higher self-rated mastication to SRH. It would not be sufficient to assess only the number of teeth as a risk factor for SRH. Although the number of teeth can be easily assessed in all questionnaire studies on oral status, similar to our study, the mastication status should also be investigated.

Our study has several limitations. First, our survey of the number of teeth and masticatory status was conducted using a questionnaire, and clinical examination was not conducted. As several reports have validated the accuracy of the correlation between clinical examination and self-report of the number of teeth and masticatory status^36,37^, we believe that our method may not have significantly influenced the results. Nevertheless, performing clinical examination for determining the number of teeth and assessing the masticatory status is desirable and preferable. Second, we did not confirm whether the patients used removable or non-removable prostheses. Individuals with fewer than 20 teeth who could bite on both sides were assumed to be using a type of prosthesis. Although it is rare, there might have been participants who experienced good mastication on both sides, even without occlusal support areas. For a more accurate survey of the combination of the number of teeth and self-rated mastication, a questionnaire on the use of prostheses should be administered in the future. The third limitation is related to the generalization of this study due to missing data. The original target sample was 8783 participants; however, the self-administered questionnaires were completed by 7447 individuals, and the data of 708 participants were excluded because of incomplete answers on SRH, number of teeth, and self-rated mastication. It is difficult to determine whether the remaining number included in the analysis is accurate. The fourth limitation is the possibility of the presence of other effective confounding factors that were not adjusted for in our model. For example, age-related changes such as menopause in women, which was not adjusted for in this study, can contribute to poor SRH^38^. Menopause is well-known to have various symptoms in women^39,40^. The risk of osteoporosis and metabolic abnormalities such as glucose metabolism disorders derived from a reduction in postmenopausal estrogen level is also well-known^39,40^. Furthermore, these diseases are well-known risk factors not only for poor SRH but also for periodontal disease that can lead to tooth loss^41–44^. Therefore, age-related changes such as menopause in women also may affect SRH. Although we adjusted for various disease conditions as a part of medical history, the confounding factors adjusted for in our model were limited.

In conclusion, the present study found that individuals with less than 20 teeth who could not bite on both sides were at risk of poor SRH compared to individuals with at least 20 teeth who could bite tightly on both sides. Furthermore, the present study found that for individuals who could bite tightly on both sides, those with less than 20 teeth were not at risk of poor SRH compared to those with at least 20 teeth. Our study suggests the importance of having at least 20 teeth and oral rehabilitation using a type of prosthesis for SRH, even if the individual has less than 20 teeth.

## Data Availability

Data cannot be shared for privacy or ethical reasons but are available from the corresponding author on reasonable request after permission of the ethics committee of Yamagata University.
